# Intellectual Property Rights in China—A Literature Review on the Public's Perspective

**DOI:** 10.3389/fsoc.2022.793165

**Published:** 2022-04-12

**Authors:** Katrin Muehlfeld, Mei Wang

**Affiliations:** ^1^FB IV—Department of Business Administration, Trier University, Trier, Germany; ^2^Chair of Behavioral Finance, WHU-Otto Beisheim School of Management, Vallendar, Germany

**Keywords:** intellectual property rights, China, public perception, patents, copyrights, trademarks

## Abstract

Despite significant advances in terms of the adoption of formal Intellectual Property Rights (IPR) protection, enforcement of and compliance with IPR regulations remains a contested issue in one of the world's major contemporary economies—China. The present review seeks to offer insights into possible reasons for this discrepancy as well as possible paths of future development by reviewing prior literature on IPR in China. Specifically, it focuses on the public's perspective, which is a crucial determinant of the effectiveness of any IPR regime. It uncovers possible differences with public perspectives in other countries and points to mechanisms (e.g., political, economic, cultural, and institutional) that may foster transitions over time in both formal IPR regulation and in the public perception of and compliance with IPR in China. On this basis, the review advances suggestions for future research in order to improve scholars' understanding of the public's perspective of IPR in China, its antecedents and implications.

## Introduction

“Property is ubiquitous” (Carruthers and Ariovich, 2004, p. 23). It is also powerful, in both the advantages it may confer to its owners and the disadvantages experienced by those who lack it. Increasingly, though, in a world characterized by means of labels such as “knowledge economy” (e.g., Powell and Snellman, 2004, p. 200) or “knowledge society” (e.g., Drucker, 1993, p. 52), in which data is hailed as the “new oil” (e.g., The Economist, 2017) and companies as well as countries strive for leadership in technologies such as Artificial Intelligence (AI), machine learning, or robotics, this property is “intellectual property” (IP). Consequently, intellectual property rights (IPRs) and their protection is a core concern of economic and political actors around the globe. It is also a highly contested issue, centering both on individual cases of possible infringement as well as more broadly on disagreements regarding the fundamental conceptualization of IPRs.

Over the past decades, China has emerged as a key factor in the global arena of IP, owing on the one hand to its economic power and ambition, and its importance for world economy. On the other hand, Chinese firms and institutions have frequently been at the center of accusations of IP violations [for an overview, see., e.g., Henningsen (2010)], as documented by media headlines such as The New York Times (2007)'s “China has a world-leading knack for churning out copies and counterfeits” or “How China's legal system enables intellectual property theft” (The Diplomat, 2020). Scholars have characterized China as “both the largest producer (International Anti-Counterfeiting Coalition, 2014) and the largest consumer of counterfeit products (Cheung and Prendergast, 2006)” (Bian et al., 2016, p. 4251), although some scholars disagree with the severity of piracy in China (Schwabach, 2008).

In the meanwhile, China has become more protective of their own IP rights. As the *Financial Times* recently reported, during the years 2019 and 2020, the Chinese government revised IP-related regulations, such as reducing the burden of proof for defendants for patent law and increasing maximum damages for copyright law infringements (Sagami, 2020). The number of IP-related lawsuits in China has increased more than three times from 2016 to 2020. Rather than dealing with potential infringements on the part of Chinese companies, as has traditionally been the case, foreign businesses in China increasingly have to be prepared to be sued by Chinese competitors. For example, a Tokyo-based company, Ryohin Keikaku, had to face a trademark lawsuit by two Chinese companies. Although the Japanese company won the case, the court process lasted for two and a half years (Sagami, 2020). On the other hand, overseas Chinese companies have gained more experience in dealing with lawsuits. A prominent example is the legal dispute between the Chinese technology company Huawei and French fashion house Chanel. In 2017, when Huawei applied for EU trademark protection for its computer hardware, Chanel opposed the bid by claiming that the logo was too similar. The EU General Court concluded that “the visual differences are significant,” and as a result, Chanel lost against Huawei after a long-running trademark fight (BBC, 2021).

As Yu (2014) pointed out, commentators often trace the root of IP-protection issues in China to factors such as Confucian culture, Marxist ideology, censorship, and lack of rule of law (Alford, 1995; Berrell and Wrathall, 2007), which are not sufficient to help us understand the rapid development of IP landscape in contemporary China. Two streams of recent research have offered deeper insights on various grounds. A first stream of research has proposed that conceptions of IPR and preferences for protection may differ less and in other ways between (lay) individuals from China and other countries such as the U.S. than previously thought (Mandel et al., 2020). To the extent that public understanding of IPR matters for a country's stance toward IPR protection—either directly *via* behavioral consequences or indirectly through interlinkages with policymakers and legislators—these insights suggest that a better understanding of cross-country variations in IPR protection (and violation) requires accounting for the cultural and historical context in which such public perceptions are embedded. A second strand of literature has indeed pointed toward the historical embeddedness and path dependence of countries' handling of IP (Peng et al., 2017), suggesting a kind of “natural” path along which IPR protection may be expected to improve over time in response to a country's economic progress and associated political and economics interactions with the “outside world.”

All in all, these observations suggest a need to consider the mutually influential relationship between the Chinese and the “world's” perspective on IPR in China, both with respect to the development of the actual IP-related regulatory framework (e.g., Peng et al., 2017) as well as the economic actors' viewpoints, such as, in particular, the perspective of the general public in China (e.g., Mandel et al., 2020). The present article seeks to provide a first step towards addressing this important issue by reviewing extant literature. In so doing, it focuses in particular on the public's perception of and attitudes toward those types of IPR, where voluntary compliance of the public is particularly essential for the effective functioning of IPR protection, that is copyrights and trademarks.

The remainder of this paper is structured as follows. First, we summarize key foundations of IPR, starting with a definition of IPR across both the industrial and the artistic domains and including a broad overview of core theoretical rationales underlying the protection of IPR. Second, we zoom in on a China-centered perspective on IPR protection. In so doing, we start by briefly sketching the development of China's IPR regulation before reviewing factors that have influenced the implementation of IPR protection in China, such as the level of economic development, culture, institutions, and third-party actors. Following recent accounts in the legal literature, in particular (e.g., Mandel, 2016), we then broaden this view by integrating literature that considers the public's perception of IPR in China as a complementary factor, accounting for both possible roles, that is, users and producers of IP-related products and services. Finally, we review studies that investigate to what extent and in which ways the Chinese context, in particular in terms of the public's perception of IPR, might be distinctive. Third, we conclude and offer directions for future research.

## Foundations Of Intellectual Property Rights

### Definition of Intellectual Property Rights

The World Trade Organization (WTO) Agreement on “Trade-Related Aspects of Intellectual Property Rights” (TRIPS) defines Intellectual Property Rights (IPRs) as “…the rights given to persons over the creations of their minds” (World Trade Organization, 2021). It further describes as a key function of IPRs that they “…usually give the creator an exclusive right over the use of his/her creation for a certain period of time.” Commonly, two broad domains covered by IPRs are distinguished: first, the industrial, and, second, the artistic domain.

First, within the industrial domain, the major two types of IPRs are patents and trademarks, both of which have to be applied for through formal procedures. *Patents* concern inventions, and, if awarded, protection usually expires after a fixed time period (often around 20 years). In contrast, *trademarks* cover distinctive signs that distinguish the products or services of one entity from those of another. If awarded, protection *via* trademarks can last, in principle, indefinitely, as long as the protected sign remains “distinctive”.

Second, *copyrights and related (*“*neighboring*”*) rights* cover literary and artistic works. They grant legal protection to the creators of such works (e.g., authors and artists), provided these works are both original and fixed in a tangible form. Originality—as understood for example within TRIPS—means that the focal piece of work was independently created and features a minimal degree of creativity. It does not have to be unique or novel. Fixed in tangible form implies that it is durable beyond a transitory time period and can thereby be perceived, reproduced or communicated. Further, copyright rights take effect automatically whenever an artistic work (e.g., a drawing) is fixed in a tangible medium (e.g., a beermat; Mandel, 2014). They require no formal application procedure and usually apply for the lifespan of the work's creator as well as an additional time period after his or her death (often around 50 years). As anyone can be the creator of a work that is covered by copyright, for example, a simple drawing on a piece of paper, or a picture taken and uploaded to a social media website, issues of copyright protection are near-ubiquitous and rest crucially on voluntary compliance by the lay public.

Economic and political actors are involved in the creation, protection, and, sometimes, violation, of IPRs in a variety of roles: First, they may be producers or creators of IP; second, they may be users of IP, and in so doing may either comply with legal regulations or engage in infringements; third, they may shape the legal framework pertaining to IPR protection as legislators or ensure its implementation as law enforcement agents.

### Theoretical Rationales

Consultation of the WTO's TRIPS-related resources suggests a consensual view of the theoretical rationales underlying the protection of IPRs: In the case of copyrights, it is argued that the “social purpose […] is to encourage and reward creative work” (World Trade Organization, 2021), while trademarks are supposed to “stimulate and ensure fair competition and to protect consumers, by enabling them to make informed choices between various goods and services” (World Trade Organization, 2021) and patents are meant “to stimulate innovation, design and the creation of technology […] social purpose is to provide protection for the results of investment in the development of new technology, thus giving the incentive and means to finance research and development activities” (World Trade Organization, 2021).

Scholarly accounts are more nuanced. Mandel (2014) distinguishes at least three different theoretical lenses through which scholars have viewed, in particular, copyrights and patents, that is, first, the incentive theory of IPRs, second, the natural rights perspective, and, third, the expressive function perspective. Furthermore, fourth, the underlying rationale for trademarks is mostly based on consumer search and information costs (Mandel, 2014, p. 266; Landes and Posner, 2003). First, the incentive theory of IPR protection is based on the idea that without adequate protection of IP, there would be market failure in the sense of too little intellectual goods being created (Landes and Posner, 2003; Mandel, 2014). Compared to most tangible goods, intellectual goods present considerably greater challenges in terms of allowing their creators to reap the benefits of their resource investments (e.g., time, financial resources, and physical input goods). Most intellectual goods are characterized by high degrees of non-excludability (i.e., non-paying individuals cannot easily be excluded from its consumption) and non-rivalry (i.e., consumption by one individual does not hinder simultaneous or subsequent consumption by another person because consumption does not deplete it as it would in case of a tangible good). That is, absent adequate IP protection, intellectual goods tend to be fairly easy to copy, share and distribute without the creator being able to control these processes and charge consumers accordingly. In addition, the outcomes of creative or inventive activities are inherently uncertain. If in case of a success at least the prospects of reaping the benefits from this outcome are certain in the sense of being protected, this is expected to stimulate creative and inventive activities. Thus, IPRs are granted to creators of intellectual goods such that they can keep other actors from copying or sharing or distributing their work without the creator's permission (Landes and Posner, 2003; Mandel, 2014). Second, the natural rights perspective draws on moral rather than economic arguments. It holds that creators of intellectual goods are, naturally and by definition, entitled to reap the benefits from their efforts and investments. Third, Mandel (2014) proposes that the expressive function perspective underlying the protection of IP draws from philosophical considerations, rather than moral or economic rationales. Instead, the creation of intellectual goods can be seen as “allowing greater human flourishing and cultural development, and should be protected for this reason” (Mandel, 2014, p. 270).^1^

## Intellectual Property Rights' Protection: A China-Centered Perspective

### Background: Development of China's Intellectual Property Rights Regulation

Modern IPR regulation in China dates back to the 1980's when China acceded to the World Intellectual Property Rights Organization (WIPRO). Initial domestic regulation included the Trademark Law in 1982, the Patent Law in 1984, and the Copyright Law in 1990 (Berrell and Wrathall, 2003). In 2001, it became a member of the WTO. Thereby, the TRIPS emerged as the relevant international framework influencing legal regulation on intellectual property rights in China (Yu, 2014), beyond the Berne Convention for the Protection of Literary and Artistic Works, the Paris Convention for the Protection of Industrial Property, and the Madrid Agreement for the International Registration of Trademarks to which it had acceded during the 1980's. The existing IP related regulations were amended correspondingly (Page, 2019; Mandel et al., 2020). However, enforcement of IPR regulation has frequently been criticized as comparatively weak, for example, relative to territories, which are historically close, such as Hong Kong or Singapore (Peng, 2013; Peng et al., 2017)—a view that is reflected also in assessments of IP producers within China and in recent years (Sun et al., 2021). Variation in the implementation of IPR protection, that is, variation that can be traced back to differences in law *enforcement* and *compliance* has been attributed by scholars to several, partly intertwined sources, which have been emphasized to different degrees by economic, cultural, actor-centered, political, and institutional approaches. The following section offers a brief overview.

### Factors Influencing the Implementation of Intellectual Property Rights' Protection in China

Scholars have increasingly recognized the importance of considering a country's IPR regime and IPR enforcement as highly contextualized in time and space. Theoretical lenses such as, in particular, the incentive theory, have undoubtedly strongly influenced IPR regulation in many countries (e.g., the U.S.; Mandel, 2014). However, the underlying reasoning is essentially decontextualized. Yet, contextual factors such as, for example, culture or politics, seem to play a particularly important role for the way a given IPR regulation is being implemented, that is, enforced and complied with (e.g., Kshetri, 2009).

First, the level of *economic development* of a country has generally been argued and found to be correlated with the strictness of IPR protection favored by and enforced in a country (e.g., Marron and Steel, 2000): IPR protection and enforcement generate both benefits and costs (e.g., Peng et al., 2017), and the balance seems more favorable for more advanced economies, as they typically have more firms relying on (highly) innovative rather than predominantly imitative business activities and tend to be more active exporters of knowledge-intensive products and services (cf. Kim and Mudambi, 2020). Recent developments in China have, consequently and in line with its increasing level of economic development, been argued to reflect progress in terms of transforming a predominantly imitation-oriented economy into an innovation-driven economy (e.g., Page, 2019). The development of Chinese IPR regulation can, thus, be seen to follow a typical path in this respect (e.g., Peng et al., 2017), even though compliance, in particular, seems to be lagging behind.

Thus, second, scholars have emphasized the importance of characteristics of a country's *culture*. In the case of China, the dominant view holds that a lack of respect for IPR is deeply culturally rooted in its religious tradition of Confucianism (Alford, 1995; cf. Kshetri, 2009, 2017). Specifically, scholars have pointed to culturally-embedded conceptions of *creativity* and culture-dependent normative assessments of *imitation* (Henningsen, 2010).

Henningsen (2010), for example, argues, that Chinese conceptions of what constitutes a creative (authentic) piece of work in arts, literature, and beyond in everyday life, in China are traditionally broader and more comprehensive, whereas analogous conceptions in most Western countries tend to be narrower and more de-contextualized. For example, to the extent that considering a piece of work as “creative” requires it to be “unique”—as is typical of contemporary conceptions in most Western societies—, this assessment implies a need to “turn a blind eye toward traces of the work's ancestors, of the models on which it is based, and of the traditions the artist was born into” (Henningsen, 2010, 19–20). Consequently, some scholars have put forward the notion of “adaptive creativity,” which emphasizes refinement and recycling of ideas, to capture more comprehensive conceptions of creativity (e.g., Keane, 2007; Henningsen, 2010).

Relatedly, in terms of normative assessments of imitation, scholars have suggested that the culturally prevailing view in contemporary China remains more positive than in many other countries. Henningsen (2010), for example, described the important role that imitation has traditionally played in the Chinese educational system—an element that is presumably fostered by the logographic written language system of Chinese language. She further argues that also in the domains of the arts and philosophy, imitation and learning tend to be viewed as inextricably linked processes, with “linmo” representing a specific technique in which artists develop their skills by explicitly copying existing (master) pieces of work. The popularity of “shanzhai,” a term of counterfeit, imitation, or parody products, is a modern manifestation of the traditional imitation culture (e.g., Page, 2019). “Shanzhai” is literally translated as “mountain fortress” or “mountain village,” referring to groups of bandits who opposed and evaded the imperial government control and accumulated contraband in their typically remote strongholds in the mountains (Page, 2019). Indeed, empirical evidence seems to support the notion that cultures, which view the individual as more closely and more comprehensively embedded in both present and ancestorial social contexts—as do collectivistic compared to individualistic cultures—are less sternly opposed to imitative practices that may infringe modern IPR regulations. For example, Marron and Steel (2000) in an empirical study of 53 countries found evidence that countries with more collectivist cultures tended to have higher rates of software piracy than those with more individualist cultures, even if controlling for the level of economic development of a country.

Yet, scholars have also pointed out that IPR protection varies considerably across countries that are presumably similar in terms of their cultural roots and conceptions of creativity and imitation (e.g., China, Hong Kong, Singapore; Peng et al., 2017). Also, countries which diverge significantly in these cultural respects nevertheless have been documented to show highly similar patterns in terms of disrespecting IPR (e.g., Peng, 2013). Finally, from a historical perspective, it seems likely that changes in national IPR regulation and implementation have typically occurred more frequently and at shorter timescales than developments of the underlying cultural dimensions (Peng et al., 2017). In sum, thus, culture alone is unlikely to account for cross-country variation in IPR implementation.

Third, conceiving of culture as an informal part of the more overarching institutional framework, scholars have recently put forward an *institution-based view of IPR* implementation and its development over time across countries (Peng et al., 2017). At the heart of this perspective is an emphasis on the incentive-determining structure of IPR institutions as “rules of the game” (comprising both regulations and their enforcement) as well as the identification of three theoretical mechanisms (Peng et al., 2017) that explain IPR compliance and IPR-related attitudes in a given country at a particular point in time, that is: path dependence, long-term processes, and institutional transitions. According to Peng et al. (2017), path dependence implies that past conditions (e.g., weak IPR protection regime) affect actors' behavior in the present even if the conditions themselves have changed (e.g., stricter legal framework for IPR protection). Aligned line with established conceptions of institutional hierarchies (e.g., Williamson, 2000), the notion of long-term processes acknowledges that institutional change may be driven by institutional isomorphism and may, especially when it involves not only formal (e.g., specific laws) but also informal institutions (e.g., normative assessments of the value of IP), stretch over long periods of time before a significant change in actors' behavior becomes noticeable (Peng et al., 2017). The institutional transitions mechanism highlights that institutional change is associated with both costs and benefits. The time horizons of these costs and benefits may not always be well-aligned and, moreover, the magnitude may vary with other contextual variables, such as, for example, the level of economic development. As a result, an analogous institutional transition (e.g., from a weak to a strict IPR regime) may take longer in some cases than others, depending on the precise contextual configuration (Peng et al., 2017).

Implicitly, by comprising processes of institutional isomorphism, the institutional perspective also points to a fourth source of variation in the implementation of IPR protection, that is the potentially important role of *third-party actors* in impacting IPR implementation and its development. Much of the literature focuses on either one of two types of actors: political and economic actors. Political actors include, in particular, other nation-states that may exert more or less directly pressure toward adapting a country's IPR regime including enforcement mechanisms (e.g., Peng et al., 2017), such as, for example, when the U.S. launched a WTO complaint against Chinese IPR practices as revealed by WTO documentation in 2018 (Mandel et al., 2020).

In terms of external economic actors, scholars have primarily investigated the role of multinational enterprises (MNEs) in influencing IPR regulation, enforcement, and compliance in countries in which they operate (e.g., Brandl et al., 2019; Kim and Mudambi, 2020). Brandl et al. (2019), for example, analyzed the influence of MNEs from advanced economies as well as of supranational organizations on a sample of 60 developing countries' that signed the TRIPS as the predominant global IPR protection standard. Considering the speed and extent with which these countries incorporated the TRIPS standards into their national IP legislations as key outcome variables of interest, they found that more extensive involvement of MNEs in the focal countries' innovation systems increased TRIPS adoption. Additionally, this relationship was positively moderated by the degree to which a country depended on supranational organizations such as the International Monetary Fund. Kim and Mudambi (2020) broadened this perspective to consider the role of “home-grown” MNEs, which may act as “keystone organizations” within domestic business ecosystems, encouraging innovation, and, thereby, compliance with IPR regulation.

Following up on the perspective advocated by Kim and Mudambi (2020), which emphasizes third-party actors within the national context, we suggest here to complement the extant actor-centered perspectives by incorporating yet another strand of literature that has, to date, not been integrated with the aforementioned research streams seeking to explain variation in IPR implementation. Specifically, in line with the propositions put forward by Mandel (2016), we argue that a more comprehensive understanding of IPR implementation, including the degree of compliance with IPR regulation, might benefit from accounting for the public's perception of IPR—both in their role as users and producers of IP-related products and services.

### Public Perception of Intellectual Property Rights in China

To start with, we offer a tentative systematization of various types of behavior that are relevant for assessing public perceptions of IPR in China and that are covered in the reviewed studies—together with an important caveat: Not only is the use of terminology not fully consistent across prior literature; additionally, an integrative analysis that unifies the different streams of literature and, thereby, also offers an overarching and comprehensive conceptual framework of all relevant IPR-related behaviors (e.g., counterfeiting, patent infringement) and speaks to the transferability of public attitudes across these different behaviors, is still lacking.

So tentatively, we point out the following conceptual distinctions. First, imitation is often conceptualized in contrast to authenticity, originality, uniqueness or genuineness (e.g., Henningsen, 2010). If used in this sense it represents a kind of umbrella term, which comprises a broad range of specific behavioral manifestations that may or may not be in violation of IPR regulation (e.g., copying products without trademark infringement). However, second, some of these manifestations are themselves also referred to as imitation in the literature. Le Roux et al. (2016), for example, developed a nuanced typology of distinct forms and modalities of imitative practices relating to established products. At the heart of their typology is the fundamental distinction between counterfeit [i.e., an “exact copy of an original item” (p. 350), also referred to as a “fake”] and imitation (with the term being used in a narrower sense to refer to a product that looks similar to some other product but does not seek to constitute an exact copy, including copycats, lookalike or me-too products). Thus, while counterfeits are unequivocally associated with IPR infringements, this is not necessarily the case for imitative products, but tends to depend on the degree of similarity with the original product. This degree of similarity, in turn, is influenced by the number of product features, which are similar, their salience, and distinctiveness. A further term related to and sometimes compared with counterfeiting and imitation is piracy, which, on the supply side, often takes the form of illegal copying of (digital) products such as software or movies. In prior research, it has also been characterized as non-deceptive counterfeiting (see, e.g., Kwong et al., 2003; Le Roux et al., 2016), in which consumers essentially act as accomplices, who are fully aware that they are purchasing non-genuine products (e.g., Bloch et al., 1993).

In sum, Le Roux et al. (2016), therefore emphasize that neither of the aforementioned broader types different behavioral manifestations (e.g., counterfeiting, imitation, and piracy) represents a homogenous category. Instead, each category comprises in itself a variety of further differentiated manifestations—and public perceptions of and attitudes toward each of them may, in principle, differ.

#### Users' Perspectives on Intellectual Property Rights

##### Attitudes Toward the Consumption of Counterfeit and Pirated Products

A burgeoning literature has begun to investigate Chinese consumers' attitudes toward counterfeit and pirated products (often luxury products, pirated software, and entertainment products), focusing primarily on the identification of moral, ethical, and socio-demographic antecedents of attitudes and purchase intentions, as consumers' attitudes and behavioral intentions crucially affect the demand side of counterfeiting. Consequently, key constructs of interests have included possible attitudinal antecedents such as integrity, moral judgement, religiosity, ethical concern, as well as socio-demographic variables like gender, age, education, household income (e.g., Kwong et al., 2003; Cheung and Prendergast, 2006; Bian and Veloutsou, 2007; Chen et al., 2018).

In terms of *theoretical basis*, many of these studies drew on Ajzen's Theory of Planned Behavior (TPB) (Ajzen, 1991; e.g., Chen et al., 2009; Phau and Ng, 2010; Jiang et al., 2019), in line with the attitudinal and intentional constructs to be explained. Frequently, either as complement or as sole conceptual basis, studies also drew from conceptualizations of ethical decision-making (e.g., Phau and Teah, 2009; Chen et al., 2018), such as, in particular social cognitive theory of moral thought and action [Bandura, 1991; Bandura et al., 1996; for applications, see, e.g., Chen et al. (2018)]. In addition, scholars found it useful to develop conceptual frameworks based on psychological theories related to individuals' coping with cognitive dissonances, their self-affirmation, moral disengagement, and moral decoupling (e.g., Chen et al., 2018). Also, several studies pursued a primarily empirical approach without explicitly or extensively drawing from particular theories (e.g., Kwong et al., 2003; Cheung and Prendergast, 2006; Bian and Veloutsou, 2007).

In terms of *methodological approach*, the majority of these studies implemented surveys—more recently usually online—, which included self-report attitudinal scales as well as sometimes hypothetical scenarios to be assessed by the respondents in their native language. The samples—gathered in China and Taiwan—mostly consisted of convenience samples of diverging sizes (e.g., Bian and Veloutsou, 2007: *N* = 295; Chen et al., 2018: *N* = 334; Chen et al., 2009: *N* = 584; Cheung and Prendergast, 2006: *N* = 1,152; Jiang et al., 2019: *N* = 412; Kwong et al., 2003: *N* = 306). In some cases, the sampling frame was restricted to a specific region or several regions (e.g., Jiang et al., 2019: Chengdu; Cheung and Prendergast, 2006: Hong Kong, Shanghai, Wuhan) or comprised purposive elements (e.g., Bian and Veloutsou, 2007: recruitment in both flea markets and high street; Kwong et al., 2003: recruitment in a district with high incidence of pirated CD sales). Offline surveys in specific locations usually managed to achieve more balanced and more systematically composed samples (e.g., Cheung and Prendergast, 2006) than online surveys. A few studies employed a fundamentally different approach. Bian et al. (2016) recently conducted a qualitative interview study with 16 Chinese consumers purposively sampled to have had prior experience with purchasing counterfeit goods.

*Findings* vary across studies, in line with heterogeneity in research questions and methodologies. Still, several insights appear to have emerged fairly robustly. First, in face-to-face settings, respondents appear to be reluctant to admit to the purchase of counterfeit products in the past or corresponding intentions for future purchases (Bian and Veloutsou, 2007). Second, demographic variables such as age, gender, or education appear to have surprisingly little predictive value and the results are often inconclusive across studies (e.g., Prendergast et al., 2002; Cheung and Prendergast, 2006; Bian and Veloutsou, 2007). In cases where demographic variables were found to be significantly related to attitudes toward or intentions to engage in IPR-violating behaviors, the findings appear to be largely tied to the specific research context. Kwong et al. (2003), for example, found that in particular younger, male individuals held favorable attitudes toward purchasing pirated CDs. Similarly, Cheung and Prendergast (2006) found gender to matter primarily in relation to specific categories of IPR-violating products with males being susceptible primarily to pirated video disks and females being more prone to purchasing counterfeit clothing and accessories. Third, constructs derived from TPB, in particular, attitudes toward violating IPR (e.g., toward software piracy), associated subjective norms and perceived behavioral control have in turn been identified as fairly good predictors of consumers' intention to engage in behavior that represents an IPR infringement (e.g., use pirated software; e.g., Chen et al., 2009). Fourth, this also applies to related constructs from the domain of moral judgment, such as moral intensity (e.g., Chen et al., 2009) and moral judgment (Chen et al., 2009, 2018; Jiang et al., 2019). Jiang and colleagues, for example, found that moral judgment, integrity, religiosity, and ethical concern were all negatively associated with (positive) attitude toward counterfeit luxury products, which, in turn, was positively related to purchase intentions of counterfeit luxury products. In a similar vein, Kwong et al. (2003), identified the social cost that an individual perceives piracy to have, his or her anti-big business attitude, the social benefit of dissemination that he or she might perceive, and the individual's ethical belief regarding IPR violation as constructs associated with the intention to engage in behavior, which at least implicitly endorses IPR-violation (purchase of pirated CD's).

Recent studies have also sought to disentangle underlying cognitive processes by analyzing mediating and moderating effects. For example, Chen et al. (2018) found that moral rationalization did not have the expected direct effect on individuals counterfeit purchase intention, but that its impact was mediated by moral judgment. Similarly, while moral decoupling did not directly affect purchase intention, it had an influence mediated by the perceived benefit from a counterfeit purchase. Overall, they concluded that even if individuals recognized the moral challenges associated with violating IPR (here: through counterfeit purchases), various mechanisms such as moral rationalization and moral decoupling might allow them to engage in moral disengagement, such that their behavioral intentions would not be inhibited.

Based on a qualitative interview study, Bian et al. (2016) complemented these findings by identifying various psychological motivations for counterfeit purchasing (e.g., enhanced self-image, “thrill of the hunt”) as well as coping strategies (“neutralization” through denial of responsibility or appealing to higher loyalties) that may help consumers address possible cognitive dissonances resulting from their IP violating behavior and ethical norms.

Finally, further insights emerge from considering the extant literature in conjunction. For example, consumers appear to find it much easier to admit to being drawn toward counterfeit products or to having purchased in the past when surveyed in online compared to face-to-face settings (cf. Bian and Veloutsou, 2007; Jiang et al., 2019). Also, while robust conclusions are not feasible, due to differences in research designs, the findings of the various studies appear to tentatively point to developments over time in the Chinese public's perception of IPR—a possibility that calls for future research to undertake systematic, longitudinal studies of such developments, their antecedents, and consequences.

##### Understanding of Intellectual Property Rights Regulations in General

Beyond individual assessments regarding one's intention to engage in or abstain from IPR violating purchases of counterfeit or pirated products, a nascent literature has begun to analyze in more comprehensive terms the knowledge about and normative assessments of IPR and IPR regulations as held by the lay public (Mandel, 2014, 2016; Mandel et al., 2020). In an online survey involving 102 Chinese university students from Yunnan, Mandel et al. (2020) confronted respondents with a series of IP-related vignettes, legal compliance questions, and further questions to collect among others basic demographic information. When comparing respondents' assessments regarding property rights in various domains (patent vs. copyright vs. personal property vs. real property), Mandel et al. (2020) found little difference across domains. Furthermore, respondents indicated in their answers to the legal compliance questions [e.g., “How important is it for people to comply with intellectual property rights?” on a scale from 0 (extremely not important) to 100 (extremely important); Mandel et al., 2020, p. 256] a clear tendency toward valuing compliance with property rights including IPR. Interestingly, this result is somewhat in contrast with the findings from the studies covered in the previous section. Most of these studies have been conducted earlier and have also investigated more concrete purchase intentions of counterfeit and pirated goods, rather than abstract normative assessments of IPR as in the study by Mandel et al. (2020). Whether this discrepancy reflects differences in the underlying samples, changing attitudes over time, or an illustration of moral decoupling is, however, an issue that needs to be addressed by future research.

#### Producers' Perspectives on Intellectual Property Rights

A scant literature has sought to assess the supply side of IPR violating products in China as well. In particular, a recent qualitative interview study by Sun et al. (2021) assessed Chinese design professionals' (managers and designers) views on IP (*N* = 49). First, they found diverging levels of IPR awareness, regarding what constitutes IPR and how it is or can be protected. In particular, individual with work experience for large companies were more knowledgeable; as were those involved in industrial compared to graphic design. Second, with respect to perceived effectiveness of IPR law enforcement, they found that most design professionals thought that IPR protection in China would benefit from further strengthening, that it tended to benefit primarily larger firms as smaller establishments lacked the required resources for building IPR protection related knowledge, and that there were considerable regional differences in the availability of IP-related support and in the strength of law enforcement, a divergence that was also found across design domains. Third, the results of Sun et al. (2021) interview study underscored the importance of ethical considerations also on the supply side, with interviewees highlighting the role of both individual ethical beliefs as well as the ethical climate within design firms and client companies.

### Distinctiveness of the Chinese Context

An important question that arises from this overview of the public perception of IPR in China is: How distinct are these results? As Chen et al. (2018) suggested, individual antecedents to IPR violations such as, for example, counterfeit purchase intentions, and underlying moral mechanisms may well differ across geographical regions and cultures around the world.

Indeed, comparative studies have documented both similarities and differences between Chinese and individuals from other countries and cultures, respectively.^2^ An early study by Lai and Zaichkowsky (1999) compared brand awareness as well as perceptions and attitudes of Chinese [from the People's Republic of China (PRC), Taiwan, and Hong Kong] and Westerners with respect to the acceptability of purchasing products that imitated renowned international brands. While production of such products was mostly deemed unethical across all four subsamples, assessment of the ethicality of consumption leaned toward acceptance. Of particular interest, and contrary to expectations, the study did not reveal any strong and consistent evidence in favor of greater across-the-board acceptance of brand imitation among any of the four subsamples (e.g., PRC). In a similar vein, a survey by Bian and Veloutsou (2007), for example, revealed that Chinese consumers valued counterfeit goods even less than British consumers. Furthermore, gender and age seemed to play a role for purchasing intention and actual behavior among British consumers but not Chinese. Participants from both countries expressed relatively low willingness to buy counterfeit goods, but there is no reliable data available regarding the external validity of such self-reported measures.

In addition, there is a fairly limited literature that has investigated public perceptions of IPR in other country contexts, without any reference to China. Pueschel et al. (2017), for example, conducted a quantitative survey in the United Arab Emirates (*N* = 86) as well as a qualitative interview follow-up study (*N* = 19), in which they analyzed counterfeit luxury product consumption by consumers in the Gulf Cooperation Council (GCC) countries. Interestingly, and in contrast to the results predominantly obtained for the Chinese context, they found age to be positively associated with the likelihood of engaging in IPR violating behavior (here: purchase of counterfeit luxury products). Older individuals were, apparently, less inhibited by risk perceptions related to inferior product performance or psychosocial factors associated with counterfeit consumption. From the qualitative interview study, it further emerged that some individuals morally rationalized their counterfeit consumption based on their (Islamic) religion. For example, purchasing counterfeit instead of original products enabled them to economize on their financial resources, enabling them to donate more and share their wealth with the poor. Particularly interesting from a comparative perspective was the also religion-based assessment of “constructive purposes of counterfeit existence, such as spreading of knowledge innovation and “know-how”” (Pueschel et al., 2017, p. 190), as this perspective is well-aligned with cultural attributions of IPR violations in the Chinese context.

Finally, it is important to note that most prior studies have focused on (intended) compliance, while public perceptions regarding the appropriateness of IP laws as such has largely been neglected. A series of studies by Mandel and colleagues aimed to fill this gap (Mandel, 2014, 2016; Mandel et al., 2020). With a survey experiment designed to test understanding of and attitudes toward various property rights (e.g., patent, copy right, intellectual vs. tangible, etc.), Mandel et al. (2020) documented that U.S. Americans generally preferred stronger property rights than the Chinese. Moreover, both Chinese and U.S. American participants considered it more acceptable to take the property for a public purpose. Compared to U.S. American students, Chinese respondents did not distinguish intellectual from tangible property. Interestingly, this result is at odds with the cross-cultural psychology literature, which had previously suggested that Chinese individuals tend to be (more) context-dependent due to their holistic cognitive style developed from the collectivistic culture (Nisbett, 2004). In light of this finding, Mandel et al. (2020) suggested that rather than assuming some IP exceptionalism in Chinese culture, it might be more appropriate to perhaps take an alternative perspective and explore further the question “Why do Americans differentiate their preferences for intellectual property rights so starkly from their preferences for other property rights?” (Mandel et al., 2020, p. 219).

Investigating more in-depth whether country-specific IP exceptionalism may exist, which countries may represent “the norm” and which ones “the exception,” what antecedents and consequences may apply, requires a systematic and large-scale assessment of the public's understanding of and attitudes toward (intellectual) property rights from a cross-country comparative perspective—a research endeavor that has not yet been undertaken but may prove stimulating to future research [cf. also Chen et al. (2018)]. Such future research should also seek to carefully delineate culture-specific versus solely country-specific effects. Indeed, although prior studies have often compared IPR-related attitudes at the country level (e.g., Song et al., 2021), there is also evidence that, even in the same country, attitudes may differ across individuals who belong to different subcultures (e.g., Pueschel et al., 2017; Sun et al., 2021), and also that cultural influences on IPR-related attitudes may transcend national borders (e.g., Song et al., 2021) due to culture being an at least partly implicit and long-run phenomenon deeply engrained in social structures and processes [e.g., different levels of social-adjustive attitude of same-nationality individuals with different background cultures; see Song et al. (2021)].

## Future Research Directions

For one, a key issue, which has emerged independently from separate strands of literature (in particular, law: Mandel, 2014; international business: Peng et al., 2017) is, therefore, the question whether there really is such a thing as “country-specific IP exceptionalism,” with a few countries representing outliers and the majority constituting “the norm.” Specifically, considering China, it has frequently been claimed that IPR violation is exceptionally pervasive and socially accepted in China. However, from different disciplinary viewpoints and based on very different types of data (Mandel, 2014: consumer survey data; Peng et al., 2017: historical accounts of IPR developments across countries), these recent studies have proposed that the implementation of Chinese IPR regulation and, in particular, the public's perspective and their compliance may actually be less categorically different from major economies such as the U.S than previously thought. Instead, such an impression of categorical difference may simply reflect an as yet incomplete understanding of the complex interplay of factors such as economic development, culture, historical background, institutional framework, third-party actors (e.g., foreign MNEs), and predominant types of business ecosystem orientation (e.g., Kim and Mudambi, 2020). For example, a recent experimental study revealed that the perceived counterfeit dominance in the market negatively impacted perceived quality and purchase intention for Anglo-Americans, but not for Chinese, a finding that was attributed to the stronger social-adjustive tendency in Asian culture (Song et al., 2021). Consequently, future research might seek to build on these studies in order to undertake a systematic and larger-scale assessment of the public's understanding of and attitudes toward (intellectual) property rights from a cross-country/ cross-culture comparative perspective. In so doing, a cross-disciplinary approach, combining different disciplinary perspectives as well as methodologies, appears particularly promising in order to bring together the aforementioned streams of research from different disciplines ranging from law to international business. Future research should ideally adopt a longitudinal perspective, explicitly capturing how public perception of IPR changes over time in the focal countries, among others in response to economic developments, internal and external political pressures, or global and local IPR-related crises (such as, for example, the Covid 19 pandemic and associated discussions regarding a vaccine patent waiver).

Second, while this article explicitly sought to review IPR in China through the lens of the “public eye,” including possible differences with public perception in other countries as well as hinting at possible mechanisms of transitions, it also became clear that most prior research even beyond the studies covered here has predominantly focused on how IPR in China has been developed influenced by “the world.” In turn, China's impact on IPR regulation and implementation, including public perceptions across other countries, has remained under-explored, despite the significant and still growing importance of China for the world economy. Therefore, future research should seek to address this complementary perspective [see, e.g., Carruthers and Ariovich (2004)].

Third, an implicit assumption in many of the reviewed studies was that more IPR protection tends to be superior both morally and economically, whereas infringements are associated with social and economic losses. However, some scholars have voiced disagreement with such over-simplified assumptions. Landes and Posner (2003) as well as Posner (The Becker-Posner Blog, 2012), for example, expressed concerns that excessive patent and copyright protection could suppress competition and creativity. Posner (The Becker-Posner Blog, 2012), for example, suggested that “to evaluate optimal patent protection for an invention, one has to consider both the cost of inventing and the cost of copying.” In case of the pharmaceutical industry, for example, Posner (The Becker-Posner Blog, 2012) argued that patent protection may be necessary due to the high cost of inventing and low cost of copying—but also cautioned, that “few other products have the characteristics that make patent protection indispensable to the pharmaceutical industry.” Instead, in other sectors, inventions may be less expensive, and first movers may often naturally gain advantages. Thus, while regulators may tend to seek to educate the public to respect IPR without considering the negative effects of excessive IPR protection, it may be crucial to distinguish different products and industries regarding the public perception of IPR. It therefore seems important to elicit public opinion IPR protection for various types of products. In particular, future studies should also investigate to what extent the perceived appropriateness of IPR protection is related to the perceived and real costs of inventing and producing particular types of products.

These considerations appear especially imperative in view of contemporary technological developments (cf. Carruthers and Ariovich, 2004). An intriguing example is the current debate about whether an Artificial Intelligence (AI)-system can be named as the inventor on a patent (Croft, 2021). Such novel cases provide great opportunities to deepen our understanding on the fundamental evaluation process because people (including legislators, inventors, experts, and so on) cannot rely on conventions and have to build up their judgment and arguments from scratch. At the same time, considerable cross-country differences may be expected, in line with heterogenous public stances toward new technologies (Rieger et al., 2021).

The call for systematically distinguishing public IPR assessments based on different product categories or sectors emerges as well from considering specifically those empirical studies that have sought to directly capture the public's perception of IPR: Most of these studies so far have focused on a specific type of product in relation to which consumers' attitudes are being assessed, such as, in particular, luxury products (e.g., Jiang et al., 2019). However, luxury products represent a special type of consumption product and it remains unclear to what extent the results for one category of product (e.g., luxury goods) can be transferred to other categories (e.g., software). Individual attitudes may well differ, depending on the type of focal counterfeit product (e.g., Chen et al., 2018; related, e.g., Cheung and Prendergast, 2006). Further, subtle differences may exist across different types of (potentially) IP-violating economic behaviors, such as, for example, the manufacturing and distribution of counterfeits, the production of pirated or copycat products (e.g., Lai and Zaichkowsky, 1999; Le Roux et al., 2016). Whether and to what degree, possibly, normative assessments of one type of such behavior (e.g., counterfeiting) are applicable to others (e.g., piracy) is another largely unresolved issue that future research should seek to address.

Fifth, similarly based on the review of those empirical studies that have directly assessed the public's perception of IPR, methodological opportunities for future research emerge. Most of these studies have used cross-sectional, self-report data capturing attitudinal assessments, whereas actual behavioral evidence is scant at best. Also, while reliance on convenience samples is understandable in terms of ensuring access to a large number of respondents, it implies that the reported findings may have been biased. More generally, issues of (self-) selection and endogeneity may be of concern, especially given the sensitivity of the subject matter (e.g., Bian and Veloutsou, 2007).^3^ Thus future research might seek to move toward larger-scale, even more ambitious data collection efforts, ideally including longitudinal as well as behavioral data, too.

Ultimately, such multifaceted, multi-method, and cross-disciplinary research may even be able to speak to broader debates on fundamental issues associated with IPR such as their role in stimulating or hindering technological progress as well as perpetuating or eroding social and economic inequalities within societies as well as across countries and regions (cf. Carruthers and Ariovich, 2004).

## Conclusion

The present article reviewed prior literature in order to consolidate insights on IPR and their protection in China. First, generally, it identified at least four major theoretical rationales underlying the protection of IPR [incentive theory, natural rights perspective, expressive function perspective, and, for trademarks, consumer search and information costs perspective, see Mandel (2014)]. Second, from a China-centered perspective on IPR and their protection, IP-related regulations have undergone significant developments over the past few decades, aligning them more closely with established standards. However, enforcement of IPR regulation and compliance appear to still lag behind somewhat due to a combination of economic, cultural, actor-centered, political, and institutional factors. Yet, tentative evidence from the reviewed literature focusing especially on the public's perspective (as opposed to experts such as, for example, regulators or IP lawyers) suggests that the Chinese public's perspective also seems to be developing in the direction of enhanced compliance, both from the viewpoint of users and producers of IP-related products and services. Finally, the review of studies regarding a potential IP exceptionalism of the Chinese culture or, more broadly, the Chinese context, revealed an inconclusive picture. While there is some evidence in support of possible fundamental differences between the Chinese and other country contexts, other studies have emphasized mechanisms (e.g., political, economic, and institutional) that may foster further transitions over time in both formal IPR regulation in China as well as in the public perception of and compliance with IPR, calling into question the very notion of country-specific IP exceptionalism. Finally, based on having summarized key insights from prior research, the present article identified issues that have remained unresolved, despite the significant insights gained from the reviewed literature, and that may constitute valuable avenues for future research.

## Author Contributions

Both authors listed have made a substantial, direct, and intellectual contribution to the work and approved it for publication.

## Funding

The publication was funded by the Open Access Fund of Trier University and the German Research Foundation (DFG) within the Open Access Publishing funding programme.

## Conflict of Interest

The authors declare that the research was conducted in the absence of any commercial or financial relationships that could be construed as a potential conflict of interest.

## Publisher's Note

All claims expressed in this article are solely those of the authors and do not necessarily represent those of their affiliated organizations, or those of the publisher, the editors and the reviewers. Any product that may be evaluated in this article, or claim that may be made by its manufacturer, is not guaranteed or endorsed by the publisher.

## Footnotes

^1^Interestingly, the degree to which these different rationales have distinctly influenced contemporary IPR regulation across the globe appears to be still visible in the specific wording used in IPR regulation and related documentation, with European accounts carrying more extensive traces of rationales other than the incentive-based theory compared, for example, to the U.S. (Mandel, 2014).

^2^Interestingly, also within China, scholars have identified differences, but a large-scale, systematic assessment is still lacking. Cheung and Prendergast (2006) in their seminal study, for example, found significant differences in respondents' assessment of ethicality, legality and after-sales service of purchases of IPR-violating products between Hong Kong, Shanghai, and Wuhan.

^3^Also, in some cases, methodological choices may require further clarification, for example, when only cases were included in the analysis of respondents who had previously bought counterfeit products whereas those who had not done so were not included (e.g., Jiang et al., 2019).
